# Microscopy of the Dental Tissues

**Published:** 1870-02

**Authors:** S. P. Cutler

**Affiliations:** Prof. of Chemistry & Histology, N. O. Dental College.


### ARTICLE II.

### *Microscopy of the Dental Tissues.*

By S. P. Cutler, M.D.,A.E.,D.D.S.

Prof. of Chemistry & Histology, N. O. Dental College.

*Concluded.*

*Physiology.*—In my last article I assumed that all the life or vital forces were apparently spent on muscular motion without which there could be no animal existence, as animal existence is intimately dependent on muscular motion. Sentient impressions may be and are felt through sensorial nerves including those of special sense, as the five senses, though no response could be given in any manner to any impression received or conceived independent of muscular motion. Thinking might be carried on but could not produce any result without muscular contraction, the latter being an effect of a cause, that cause the direction of a force held in reserve.

Without muscular motion there could be no expression, no utterance; whether through the vocal organs or manual acts, all the same. In all the lower animals instincts of self preservation and reproduction are the chief ends to be accomplished, and are the prime motives of their existence. Man possesses all these, and in addition those of intellectual and moral faculties, which in the higher walks of cultivated life enable him to enjoy both the higher and lower attributes of life. In both man and animals muscular motion accomplishes or does all the work of animal life. Chemical agencies, by this term meaning both agents and actions, furnish motive power for muscular action, as they do not act within themselves but have to be acted on by some outside force sent to them independent of any innate consciousness indwelling in the muscles themselves, as already stated. As a muscle acts there is a greater amount of vital force generated there by increased influx of circulation and consequent oxidation.

This increase of force, per se, does not directly impart additional energy or power, but indirectly, through the brain and spinal column, by first being sent up through the sensorial nerves from the muscle, thence back through the outer nerve to the muscle as additional force, not only to the muscle alone but the entire organism in common, the greatest amount being spent where the greatest demand exists, as in the case of muscular activity, which is the point of greatest disturbance, demanding fresh supply of force.

No work is done without expenditure of force, even thinking requiring consumption of carbon, and, as the Germans say of phosphorus, *uno phosphor kine gideankie*, or, without phosphorus no thought. Phosphorus being more readily oxidisable, an excess of it is found in the brain and nerves over other tissues in consequence. All the forces then in organic life were in the first instance, preexistent in the inorganic elements that enter into organisms, together with free oxygen that enters and combines with all the oxidisable elements, the act of combining being the source of developing force, the actual force, as developed, coming mainly from the oxygen that enters the system. The latent caloric of the oxygen by combining, being set free in the form of sensible heat, then vital force. This force is given up by the oxygen in consequence of its lessened capacity, resulting from condensation of oxygen in its union with carbon and other elements though mainly carbon.

In the union of oxygen with carbon, the volume of oxygen being lessened, its capacity for heat is lessened in proportion, and a proportionate amount of heat force set free. In making these estimates, it must be taken into consideration that there are some losses of force resulting from a radiation from every portion of the tissues, and given up to surrounding media. Wherever there is friction there is heat force generated, then again it requires force to produce friction; and necessarily, a certain amount of force is expended in producing friction, as in the circulatory and respiratory movements, the friction of the blood upon the vessels, the air upon the air passages, muscular fibres among themselves, and upon surrounding tissues, all require expenditure of force to produce these frictions, as friction depends on motion and motion is produced by force. In turn friction develops force to a given extent, not equal to the amount expended to bring it about.

Force then may be regarded as being derived from outside influences being brought to bear upon the organism by chemical union or combination constantly taking place throughout the organism. The antagonisms of surrounding media with the organism are exhaustive influences constantly varying in temperature, absorbing heat force by radiation from the body's surface, and exhalations from the lungs of watery vapor and carbonic acid, taking out with them a large amount of caloric, thereby constantly lowering the internal temperature, as the surface does the surface temperature. No forces in the body are created, or lost, or annihilated, they are only developed or transformed from static to dynamic conditions, and from dynamic in turn to static, first derived from the static oxygen of the atmosphere, and returned to the static atmosphere by or through the agency of carbonic acid and watery vapor.

Without chemical affinity in the organism no life manifestations could possibly take place, as there could be no force developed, life manifestations being the resultant of dynamical force, also static force held in the brain as reserve ready to become dynamic by direction of will. All forces, whether in the organism or out of it, anywhere in the cosmical universe, are mutually convertible, none being created or destroyed, all phenomena depending on mutual action and reaction of matter and force.

Wherever there is an increase of force at any point there is disturbance, and this is produced at the expense of the surrounding elements which have given up the same amount of force that has been manifested by the disturbance, measure for measure, under all circumstances, the tendency being towards equilibration, which never precisely takes place. In order to first develop life force from the inorganic kingdom, solar influence is requisite in order to cause disturbance and give new directions to preexisting dormant, inorganic forces, otherwise there never could be life manifestations on the earth, all would be silent inertia and the earth would fall to absolute zero on its surface.

All undirected forces act in the direction where least resistance is offered, either in or out of the organism. This is strikingly illustrated by the action of electricity or galvanism, either in the clouds or galvanic batteries, the charge seeks the easiest route, such is the case with the telegraph also, it is easier for the force to follow the wires than to leave it, because wire is a better conductor than the surrounding media, offering less resistance.

This is the case with conducting nerves carrying vital force from the brain to the muscles, a certain amount being lost on the route, as is the case with telegraphic wires. Whether nerve force be a subtle fluid or only an effect of matter without any actual flow of something through the nerves, is yet an unsettled problem, as is the case of magnetic conductors. Molecular disturbance constitutes the recognized phenomena in either case, as no actual fluid has ever been recognized.

The exterior and interior of muscles are supposed to be in opposite states of polarity, simulating a magnetic battery. If such be true, oxidation in the muscles themselves would be facilitated, or, these opposite polarities may be the result of oxidation in the living animal though not so in the dead. Whether force will be regarded as an entity independent of matter or subservient to its control in any way is yet an unsettled point. We find it only in connection with living organisms, leaving when death take place.

Life then is the result of antagonistic forces culminating in the living organisms, resulting in death on the one hand and reproduction on the other. Life and death are continually shaking hands at every point in the living animal, and whenever these mutual exchanges cease, death or inertia is the consequence, life phenomena being exchanged for inorganic, molecular phenomena.

---

